# Gene specific therapies – the next therapeutic milestone in neurology

**DOI:** 10.1186/s42466-020-00075-z

**Published:** 2020-09-08

**Authors:** David Brenner, Albert C. Ludolph, Jochen H. Weishaupt

**Affiliations:** 1 Department of Neurology, University of Ulm, Ulm, Germany; 2 Division of Neurodegenerative Diseases, Neurology Department, University Medicine Mannheim, Mannheim, Germany; 3 German Center for Neurodegenerative Diseases (DZNE), Ulm, Germany

## Abstract

Gene selective approaches that either correct a disease mutation or a pathogenic mechanism will fundamentally change the treatment of neurological disorders. Basically, gene specific therapies are designed to manipulate RNA expression or reconstitute gene expression and function depending on the disease mechanism. Considerable methodological advances in the last years have made successful clinical translation of gene selective approaches possible, based on RNA interference or viral gene reconstitution in spinal muscular atrophy (SMA), Duchenne muscular dystrophy (DMD), and familial amyloid polyneuropathy (FAP). In this review, we provide an overview of the existing and coming gene specific therapies in neurology and discuss benefits, risks and challenges.

## Background

After the ground-breaking therapeutic advances in neurovascular and chronic inflammatory CNS diseases in the previous decades neurology reaches the next therapeutic milestone. Gene-specific approaches that either correct a genetic defect directly or compensate a disease mechanism will dramatically change the treatment of both genetic and sporadic neurological diseases. The ultimate gene specific therapy would be the direct, precise and permanent correction of the DNA defect. Advancement of genome editing tools and vector platforms might make this dream come true one day. Meanwhile, indirect gene-specific strategies have been continuously developed and are now available to effectively compensate the effects of mutations or pathomechanisms. Fundamentally, three different approaches are used in practice today:

1. RNA interference by antisense oligonucleotides (ASOs) or small interfering RNAs (siRNAs) to manipulate stability, splicing and/or translation of the target mRNA.
2. Splice modification by small molecules leading to in- or exclusion of a selected exon.
3. Gene (transfer) therapy - viral delivery of an exogenous DNA encoding a healthy gene copy or a regulatory RNA (miRNA or shRNA) to influence expression of the target mRNA.

Gene mutations cause a partial or complete loss of function (LoF), a gain of toxic function (GoF) or a combination of both. If the prevailing disease mechanism is a LoF the therapeutic aim is to restore the expression of the affected protein product (“gene reconstitution or replacement”). In the case of a GoF the goal is to reduce the expression of the mutant protein product (“gene silencing”). Technically, gene delivery therapies and ASOs both can be designed to down- or upregulate expression of the target gene/mRNA. So far, gene-specific therapies have been approved for three neurological indications: Spinal muscular atrophy (SMA), Duchenne muscular dystrophy (DMD), and familial amyloid polyneuropathy (FAP). There is a constantly growing number of preclinical and clinical trials testing gene-specific approaches in other neurological diseases. This review provides a comprehensive overview of the principles of and the latest developments in gene specific therapies and illustrates the risks and challenges coming with them.

## Types of gene selective therapies

### ASOs

**Fig. 1:**
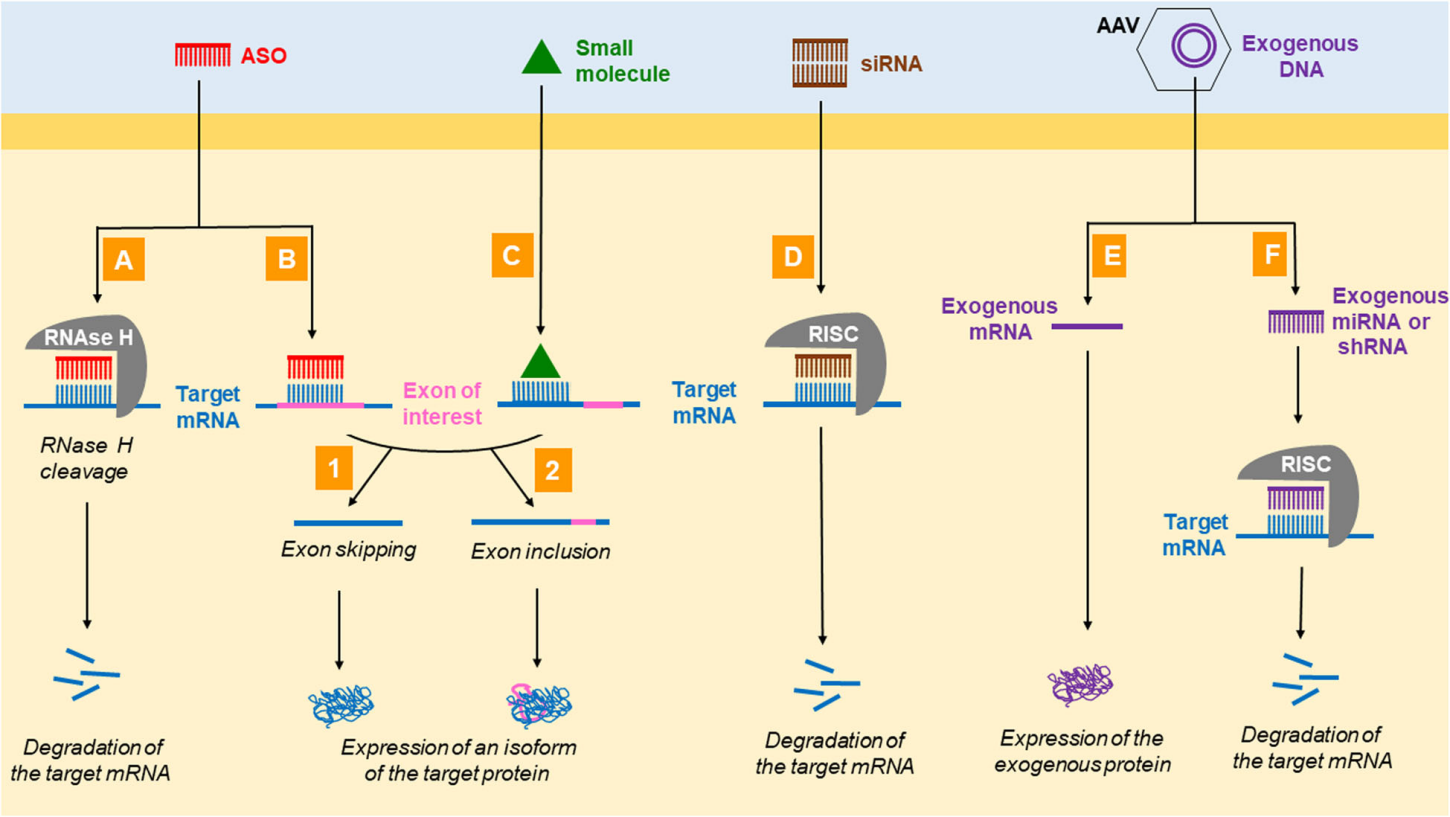
Mechanisms of gene-specific therapies against neurological diseases used in clinical practise and tested in clinical trials. Gene-specific therapy with ASOs (A, B), small molecules (C), siRNA (D) or viral gene transfer (E, F). (A) Binding of the ASO to the target mRNA leads to RNase H-mediated degradation of the target mRNA. (B) ASO targeting the splice site of a specific exon to mediate exon skipping (1) or exon inclusion (2). Consequently, an isoform of the target protein is expressed. (C) Small molecule targeting the splice site of the exon to mediate exon skipping (1) or exon inclusion (2). Accordingly, an isoform of the target protein is expressed. (D) The target mRNA is degraded after binding to the complementary siRNA that is part of the RISC complex. (E) Viral delivery of exogenous DNA, which codes for the mRNA of choice (gene reconstitution). (F) Virally introduced exogenous DNA, which encodes a miRNA or shRNA that mediates the degradation of the target mRNA

In ASO therapies, the expression of a specific mRNA can be modulated in a variety of ways by a short complementary sequence of DNA oligonucleotides. The therapeutic potential of ASOs has been known for decades, but clinical translation has only become possible through structural optimization (so-called “second-generation” ASOs) that led to improved stability (improved nuclease resistance), affinity, cellular uptake and consequently effectiveness, as well as reduced immunogenicity and toxicity [1, 2]. Mechanisms of action that are already used or tested in daily clinical practice or therapeutic studies are RNase H-mediated degradation of the target mRNA (Fig. 1A) and manipulation of alternative splicing to include or exclude (un)wanted exons (Fig. 1B). The modulation of miRNAs by ASOs to alter expression of target mRNAs is more complex and still in the preclinical stage. The ASO can be designed to be allele selective (only mutant mRNA is targeted) or nonselective (both mutant and wild type mRNA is targeted). Since the ASO generation available today does not cross the blood-brain barrier, the application must be carried out by intrathecal injection in the case of CNS disorders. A possible strategy to sufficiently target certain tissues after systemic administration consists in coupling the ASO to a specific conjugate substance (e.g. GalNAc [3]) or enveloping the ASO in a vector (e.g. lipid nanoparticle [4]). In contrast to virus-mediated gene correction, which, according to the current state of knowledge, is permanent after a single administration, ASOs have a limited half-life and need to be administered on a regular basis. This can be regarded both as an advantage and a disadvantage: Repeated lumbar punctures represent a burden for the patient, but the application is CNS-specific and treatment can be stopped in the case of severe adverse effects.

### siRNAs

In contrast to an ASO that is made up of a chemically modified DNA single strand, siRNA consists of a 20–25 bp short RNA double strand. Like an ASO the nucleotide sequence of the sense strand of the siRNA is complementary to the target mRNA. Incorporation of the sense siRNA strand into the RNA-induced silencing complex (RISC) leads to the degradation of the complementary RNA after its binding to the RISC [5] (Fig. 1D). Due to the limited bioavailability of naked siRNA following systemic administration this approach requires a vector. In the case of the first instance of a clinically applicable siRNA therapy, Patisiran, a lipid nanoparticle serves as vector [6].

### Small molecules

RNA splicing with in- or exclusion of (un)wanted exons can also be manipulated by small molecules, which target splice sites (Fig. 1C) and exhibit a comparably good biodistribution and tissue penetrance [7]. Risdiplam, which leads to selective inclusion of exon 7 in the *SMN2* mRNA to treat SMA, is the first approved drug based on this approach (see below).

### Vector-based gene therapy

Constant optimization of delivery, transgene expression efficacy and safety of AAVs in the recent years now facilitate successful clinical translation of gene transfer therapies [8]. In virus-mediated in vivo gene therapy a viral vector, usually a non-replicating natural or engineered AAV capsid, is used to deliver a fully functional copy of a gene including a promoter and polyadenylation signal into the nucleus of the patient’s cells without integration in the patient’s genomic DNA [9]. The transferred gene either encodes a functional protein to compensate a LoF (“gene replacement”) (Fig. 1E) or a regulatory RNA (miRNA or shRNA) to downregulate the expression of the target mRNA by RNA interference in order to counteract a GoF (“gene silencing”) (Fig. 1F). The pharmacokinetics and tropism depend on the properties of the natural or engineered AAV subtype. The choice of the promoter decides if the exogenous gene is constitutively or conditionally expressed and in which tissue it is expressed. Since tissue barriers limit the bioavailability of the vector, in some instances it needs to be injected into the CSF or directly into the target tissue (muscle or CNS by stereotaxis). The great advantage of gene delivery therapies over ASO, siRNA or small molecule approaches is the durable expression and efficacy. A potential severe disadvantage is that treatment cannot be terminated and unwanted effects may persist.

### In vivo genome editing

With CRISPR/Cas9 (Clustered Regularly Interspaced Short Palindromic Repeats), TALEN (Transcription activator-like effector nuclease) and ZFN (Zinc-finger nuclease) various gene editing methods are used in preclinical models of neurological diseases. These approaches are potentially suitable for the direct, precise and permanent correction of the DNA defect what might once become the ideal therapy. While TALEN and ZFN recognize and bind the target genomic DNA sequence directly through protein/DNA interaction, CRISPR-Cas nucleases are guided by RNA (RNA/DNA base pairing). The nucleases are designed to recognize and cut the target DNA sequences and cause double-strand breaks that are repaired by the cell’s DNA repair systems through non-homologous end joining or homology-directed repair, given the presence of a donor DNA template. Either the nuclease, its mRNA or its gene must be delivered to the target cells by a vector system that can be a plasmid, virus or nanoparticle. In either case, many technical hurdles remain to be overcome until these therapies are safe and efficient enough to treat patients [10]. Nevertheless, the pipelines of several pharma consortia already mention preclinical testing of genome editing strategies for various indications including Huntington’s disease and familial amyloid polyneuropathy.

## Present and future gene specific therapies

A myriad of gene specific therapies based on ASOs, siRNA, small molecules and gene transfer is currently being tested in a preclinical and clinical trials by academia and pharma industry. In the following, we highlight the most relevant developments in various neurological diseases while focusing on therapies having already reached the clinical trial phase (see also Table 1).

**Table 1:** Gene specific therapies approved for treatment or tested in clinical trials

Disease	Drug name	Sponsor	Target or reconstituted gene	Type of therapy	Route of administration	Mode of action	Corr. to Fig. 1	Current status	Trial(s)
**Motoneuron disorders**									
**SMA**	Nusinersen	Biogen/Ionis	*SMN2* mRNA	ASO	Intrathecal	Exon inclusion	B	Approved	NCT02193074, NCT02292537
	Onasemnogene abeparvovec	AveXis/Novartis	*SMN1* gene	rAAV9	Intravenous	Gene reconstitution	E	Approved	NCT03306277
	Risdiplam	Roche	*SMN2* mRNA	Small molecule	Oral	Exon inclusion	B	Approval expected soon	NCT02908685, NCT02913482, NCT03032172, NCT03779334
	Branaplam	Novartis	*SMN2* mRNA	Small molecule	Oral	Exon inclusion	B	Phase 1/2	NCT02268552
**SOD1-ALS**	Tofersen (BIIB067)	Ionis/Biogen	*SOD1* mRNA	ASO	Intrathecal	RNase H	A	Phase 3	NCT02623699
**C9-ALS**	BIIB078	Ionis/Biogen	*C9ORF72* mRNA	ASO (Mutation specific)	intrathecal	RNase H	A	Phase 2	NCT03626012
**Sporadic ALS**	BIIB100	Biogen	*XPO1* mRNA	ASO	Intrathecal	Probably RNase H	A	Phase 1	NCT03945279
**Movement disorders**									
**HD**	Tominersen (RG6042)	Ionis/Roche	*HTT* mRNA	ASO	intrathecal	RNase H	A	Phase 3	NCT03761849
	WVE-120101 WVE-120102	Wave Life Sciences/Takeda	*HTT* mRNA	ASO (Mutation specific)	intrathecal	RNase H	A	Phase 1/2	NCT03225833 NCT03225846
	AMT-130	UniQure	*HTT* mRNA	AAV5	intrastriatal	Expression of miHTT/RISC	F	Phase 1/2	NCT04120493
**iPD**	VY-AADC01	Neurocrine Biosciences/ Voyager Therapeutics	*AADC* gene	AAV2	intrastriatal	Gene reconstitution	E	Phase 1	NCT01973543
	AAV-hAADC-2	Jichi Medical University	*AADC* gene	AAV2	intrastriatal	Gene reconstitution	E	Phase 1	NCT02418598
**iPD and LRKK2-PD**	BIIB094	Ionis/Biogen	*LRRK2* mRNA	ASO	intrathecal	Probably RNase H	A	Phase 2	NCT03976349
**GBA-PD**	PR001	Prevail Therapeutics	*GBA1* gene	AAV9 gene therapy	intracisternal	Gene reconstitution	E	Phase 1/2	NCT04127578
**Dementia**									
**Sporadic AD**	BIIB080	Ionis/Biogen	*MAPTR* mRNA	ASO	Intrathecal	RNase H	A	Phase 1/2	NCT03186989
**Muscle diseases**									
**DMD**	Eteplirsen	Sarepta Therapeutics	*Dystrophin* mRNA	ASO (Mutation specific)	Intravenous or subcutaneous	Exon 51 skipping	B	Approved (only FDA)	NCT02255552
	Golodirsen	Sarepta Therapeutics	*Dystrophin* mRNA	ASO (Mutation specific)	intravenous	Exon 53 skipping	B	Approved (only FDA)	NCT02500381
	Multiple	Sarepta therapeutics, Nippon Shinyaku (NS) Pharma, Daiichi Sankyo, Wave Life Sciences	*Dystrophin* mRNA	ASO (Mutation specific)	Intravenous or subcutaneous	Exon skipping of exon 45, 52 or 53	B	Phases 1–3	NCT02500381, NCT04004065, NCT03675126, NCT03167255, NCT02667483, NCT03508947
	Ataluren	PTC Therapeutics	*Dystrophin* mRNA	Small molecule	oral	“Read-through”		Approved	NCT01826487
	Multiple	Sarepta Therapeutics, Pfizer, Solid Biosciences	Micro/Mini *Dystrophin* gene	AAV	intravenous	Gene reconstitution	E	Phase 1–2	NCT03375164, NCT03769116, NCT03368742, NCT03362502, NCT03333590
**LGMD2D**	MYO-102	Sarepta Therapeutics	*SGCA* gene	AAVrh74	intraarterial	Gene reconstitution	E	Phase 1/2	NCT01976091
**LGMD2E**	MYO-101/ SRP-9003	Sarepta Therapeutics	*SGCB* gene	AAVrh74	intravenous	Gene reconstitution	E	Phase 1/2	NCT03652259
**Pompe disease**	SPK-3006	Spark Therapeutics	*GAA* gene	rAAV9 g	intravenous	Gene reconstitution	E	Phase 1/2	NCT04093349
	AAV2/8LSPhGAA	Asklepios Biopharmaceutical	*GAA* gene	AAV2/8	intravenous	Gene reconstitution	E	Phase 1/2	NCT03533673
**AD-CNM and XL-CNM**	IONIS-DNM2–2.5_Rx_ (DYN101)	Ionis pharmaceuticals/ Dynacure	*DNM2* mRNA	ASO	intravenous	RNase H	A	Phase 2	NCT04033159
**XL-CNM**	AT132	Audentes Therapeutics	*MTM1* gene	AAV8	intravenous	Gene reconstitution	E	Phase 1/2	NCT03199469
**Polyneuropathies**									
**FAP**	Inotersen	Ionis/Akcea Therapeutics	*TTR* mRNA	ASO	subcutaneous	RNase H	A	Approved	NCT01737398
	AKCEA-TTR-LRx	Ionis/Akcea Therapeutics	*TTR* mRNA	ASO	subcutaneous	Probably RNase H	D	Phase 3	NCT04136184
	Patisiran	Alnylam Pharmaceuticals	*TTR* mRNA	siRNA	intravenous	RISC	D	Approved	NCT01960348
	Vutrisiran	Alnylam Pharmaceuticals	*TTR* mRNA	siRNA	subcutaneous	RISC	D	Phase 3	NCT04153149
**CTM1A**	scAAV1.tMCK.NTF3	Nationwide Children’s Hospital	*NTF3* gene	scAAV1	intramuscular	Gene reconstitution	E	Phase 1/2	NCT03520751
**GAN**	scAAV9/JeT-GAN	National Institute of Neurological Disorders and Stroke (NINDS)	*GAN gene*	scAAV9	intrathecal	Gene reconstitution	E	Phase 1	NCT02362438
**Other neurological disorders**									
**LHON**	GS010	GenSight Biologics	*MT-ND4* gene	AAV2	intravitreal	Gene reconstitution	E	Phase 3	NCT02652780, NCT02652767, NCT03293524
**Fabry disease**	ST-920	Sangamo Therapeutics	*GLA* gene	rAAV2/6	intravenous	Gene reconstitution	E	Phase 1/2	NCT04046224
	FLT190	Freeline Therapeutics	*GLA* gene	AAV8	intravenous	Gene reconstitution	E	Phase 1/2	NCT04040049
**CLN2**	AAVrh.10CUCLN2	Weill Medical College of Cornell University	*CLN2* gene	AAVrh.10	intracranial	Gene reconstitution	E	Phase 1/2	NCT01414985, NCT01161576
**CLN3**	AT-GTX-502	Amicus Therapeutics	*CLN3* gene	scAAV9	intrathecal	Gene reconstitution	E	Phase 1/2	NCT03770572
**CLN6**	AT-GTX-501	Amicus Therapeutics	*CLN6* gene	scAAV9	intrathecal	Gene reconstitution	E	Phase 1/2	NCT02725580

### Motor neuron disorders

***Spinal muscular atrophy*** (SMA) is caused by homozygous LoF mutations in the *SMN1* gene that is ubiquitously expressed in the body. The age of onset and the severity of the phenotype (SMA type 1–4) depend on the copy number of the homologous *SMN2* gene. The mature *SMN2* mRNA differs from the *SMA1* mRNA by exclusion of exon 7 following alterative splicing, which results in instability and reduced amounts of the protein product. Two gene specific therapies have been developed that mediate the inclusion of exon 7 into the mature *SMN2* mRNA: the ASO Nusinersen (Fig. 1B) and the small molecule Risdiplam (Fig. 1C). Nusinersen (NCT02193074 [11], NCT02292537 [12]) is administered intrathecally every 4 months after a 1-year dosing phase. Since its approval it has revolutionized the treatment of infantile- and later-onset SMA [13]. Meanwhile there is robust evidence that adult patients with SMA also benefit from a therapy with Nusinersen [14]. Contrary to Nusinersen, Risdiplam is a small molecule and is given orally. It could in principle reach all tissues of the body. In a phase 3 study it has shown a similar clinical effectiveness as Nusinersen for SMA type 1 (i.e. infantile SMA) and is also effective in the other SMA types (NCT02908685, NCT02913482, NCT03032172, NCT03779334). Its approval for all forms of SMA is expected soon. With Branaplam another small molecule inducing exon 7 inclusion is being tested in a phase 1/2 trial (NCT02268552). The third gene specific therapy approach against SMA aims at restoring SMN1 expression through AAV9-mediated delivery of a functional SMN1 gene copy under control of a CMV promoter (Onasemnogene abeparvovec-xioi [Zolgensma]) (Fig. 1E). Onasemnogene abeparvovec-xioi, which is administered i.v. as a single dose during the first 2 years of life, has shown an excellent therapeutic effect and a sufficient safety profile in infants with SMA type 1 (NCT03306277) [15]. However, a trial with intrathecal administration of a single dose of Onasemnogene abeparvovec-xioi was stopped by the FDA after a preclinical study had revealed dorsal root ganglia (DRG) mononuclear cell inflammation in non-human primates. The drug has meanwhile been approved for i.v. administration by the FDA and EMA.

Nucleocytoplasmic translocation of TDP-43 with formation of cytosolic pTDP-43 inclusions that might cause a nuclear LoF and cytoplasmic GoF toxicity is the prevailing neuropathology in ***sporadic ALS.*** The propagation of TDP-43 pathology in the CNS correlates with the spreading of the clinical symptoms [16]. Corroborating the significance of TDP-43 in ALS, mutations in its gene (*TARDBP*) cause genetic forms of ALS [17, 18]. Consequently, the TDP-43 pathology appears a reasoned pharmacological target. However, the reduction of TDP-43 expression itself is not a suitable therapeutic approach, since TDP-43 is physiologically essential. Mice with homozygous *Tardbp* deletion are not viable and those with heterozygous KO develop an ALS-like phenotype [19]. Therefore, approaches must aim at reducing the pTDP-43 inclusions or compensate for the loss of physiological function of TDP-43. Following this thought, an ASO approach has been developed to downregulate the *XPO1* mRNA (BIIB100), which codes for the protein Exportin 1. Exportin 1mediates the nuclear export of many proteins containing nuclear export signals including TDP-43 [20] (Fig. 1A). Thus, *XPO1* inhibition is supposed to reduce nucleocytoplasmic translocation and cytosolic aggregation of TDP-43 [21]. The recruitment of sporadic ALS patients for a respective phase 1/2 study has just started at the end of 2019 (NCT03945279).

Intermediate-length polyglutamine expansions in the *ATXN2* gene are associated with an increased risk of ALS. TDP-43 mutant mice with *Atxn2*-KO or treated with ASOs downregulating *Atxn2* show a reduction of phosphorylated TDP-43 inclusions and a dramatically increased lifespan [22] (Fig. 1A). Consequently, an ASO downregulating human *ATXN2* is being developed towards a clinical trial in sporadic ALS patients.

An intronic hexanucleotide repeat expansion in *C9Orf72* and missense mutations in *SOD1* are the most frequent causes of genetic ALS *(****C9-ALS*** and ***SOD1-ALS****)* in Europe [23]. It is supposed that their pathogenicity is predominantly based on a GoF toxicity, although a LoF aspect is also discussed for *C9Orf72* mutations. After most promising results in mutant disease models, ASOs downregulating the expression of *SOD1* (Tofersen/BIIB067/IONIS-SOD1_Rx_; allele unselective) and *C9Orf72* (BIIB078/IONIS-C9_Rx_; allele selective) through RNase H mediated mRNA degradation are now being tested in clinical trials (Fig. 1A). The trial testing IONIS-SOD1_Rx_ is presently in phase 3 (NCT02623699); an interim analysis of phase 2 has raised hope for a positive study outcome [24]. The IONIS-C9_Rx_ trial is currently in phase 2 (NCT03626012), there are no interim results available yet. Further, another consortium is developing an allele-selective silencing ASO towards clinical translation for C9-ALS and -FTD. AAV mediated gene delivery of DNA coding miRNA or shRNA that downregulate *SOD1* mRNA showed excellent efficacy in mutant hSOD1 rodent or primate models [25] (Fig. 1F). Consequently, an AAV9-*SOD1*-shRNA candidate is being developed towards clinical translation by another consortium.

### Movement disorders

The current drug therapy of **idiopathic Parkinson’s disease** (iPD) aims at restoring CNS dopamine levels. Two sponsors are currently testing MR-guided intraputaminal AAV2-delivered *AADC* gene transfer in iPD patient in phase 1/2 trials (NCT02418598, NCT01973543). The *AADC* gene encodes the aromatic L-amino acid decarboxylase responsible for converting Levodopa to Dopamine. An interim analysis of NCT01973543 has shown an increase in enzyme expression and dose dependent clinical improvements [26]. While this approach might prolong the time of symptom control it is probably – like current dopaminergic drugs – incapable of influencing disease progression. Alpha-synuclein (a-syn) aggregations are the predominant neuropathology of iPD; its propagation in the CNS correlates with the spreading of the clinical symptoms [27]. The significance of a-syn in iPS is further supported by the findings that multiplications, mutations, and single nucleotide polymorphisms in the *SNCA* gene, encoding the alpha-synuclein protein, either cause or increase the risk for iPS. Cole and colleagues developed an ASO that downregulates the *SCNA* mRNA after intraventricular application. It efficiently reduced seeded a-syn inclusion load in wildtype mice and rats treated with exogenous preformed a-syn fibrils [28] (Fig. 1A). This therapy has not yet reached the clinical trial phase. Another approach targeting the a-syn pathology consists in the downregulation of *LRRK2*. Missense mutations in *LRRK2* that lead to a GoF are a frequent cause of genetic PD [29, 30] and certain polymorphisms in *LRRK2* locus modulate the risk for iPD [31]. In addition, increased LRRK2 protein activity in dopaminergic neurons in post-mortem tissue of iPD patients seems to drive the α-syn pathology [32]. Thus, downregulation of LRRK2 appears an interesting therapy strategy to alleviate α-syn pathology. Indeed, ASOs reducing expression of the *LRRK2* mRNA diminished fibril-induced seeding of a-syn inclusions in wildtype mice [28] (Fig. 1A). Consequently, a pharma consortium is testing an ASO targeting the human *LRRK2* mRNA (BIIB094/ION859) in a phase 2 trial in PD patients with or without *LRRK2* GoF mutations (NCT03976349). In case the strategies reducing a-syn pathology are clinically effective they might also be tested in other synucleinopathies, such as multiple system atrophy (MSA) and Lewy body dementia (LBD).

Mutations in the *GBA1* gene, which presumably lead to a LOF, are the most frequent genetic contributor to PD pathogenesis (***GBA-PD***) [32]. *GBA1* encodes the enzyme beta-glucocerebrosidase, which is required for the disposal and recycling of glycolipids. Accumulation of glycolipids leads to lysosomal dysfunction that in turn exacerbates lysosomal accumulation of a-syn. Recently, a phase 1/2 trial has been launched in patients with a pathogenic *GBA1* mutation (PD-GBA) testing a gene reconstitution therapy where a *GBA1* gene copy is delivered by an AAV9 (NCT04127578) (Fig. 1E). The drug is administered intracisternally as a single dose. A phase 1/2 trial testing the same therapeutic in patients with neuropathic Gaucher disease, that is caused by biallelic LoF mutations in the same gene, is expected to start soon. Further, the same sponsor announces the development of an approach combining *GBA1* gene transfer and *SCNA* knockdown for treatment of synucleinopathies in general.

A CAG repeat expansion in the *HTT* gene that encodes the Huntingtin protein causes ***Huntington’s disease*** (HD). After most promising results in preclinical rodent studies [33], several gene-specific therapeutic strategies are being tested in clinical trials in HD patients. In NCT03761849, currently in phase 3, an allele unselective ASO that downregulates pan-*HTT* mRNA (Tominersen/RG6042/IONIS-HTT_Rx_) is administered intrathecally (Fig. 1A). An interim analysis has demonstrated successful target engagement in that the HTT protein was reduced by 40% on average in the CSF of HD patients [34]. In NCT03225833 and NCT03225846, currently in phase 1/2, allele specific ASOs (targeting a prevalent SNP) selectively downregulate the mutant *HTT* mRNA after intrathecal adminstration (Fig. 1A). Third, in NCT04120493, currently in phase 1/2, an AAV5 vector is used to deliver a gene encoding a miRNA that blocks the HTT mRNA (*miHTT*). This vector is administered by intrastriatal injection (Fig. 1F).

### Dementias

Tau and beta-amyloid aggregates are the neuropathological hallmarks of **sporadic*Alzheimer’s disease*** (AD). Mutations in the amyloid precursor protein gene *APP* cause genetic AD through a GoF mechanism. However, beta-amyloid immunotherapy has been largely disappointing in clinical trials so far, questioning beta-amyloid as the ideal drug target, at least in progressed AD cases. According to autopsy and Tau-PET imaging studies spreading of Tau pathology seems to be highly correlated with disease progression [35, 36], which raises hopes for a successful clinical translation of Tau targeted therapies. Genetic deletion or ASO-mediated downregulation of the *MAPT* mRNA (encoding Tau protein) alleviated neuropathology and clinical symptoms in genetic AD mouse models. Consequently, an ASO designed to downregulate the *MAPT* mRNA is being tested in AD patients in a phase 1/2 clinical trial (NCT03186989) (Fig. 1A). If this strategy proves successful it could be also tested in other tauopathies, such as the atypical Parkinson syndromes supranuclear palsy (PSP) and corticobasal syndrome (CBS).

***Sporadic frontotemporal dementia*** (FTD) is neuropathologically associated with TDP-43, Tau or FUS protein pathology that each is considered the cause of the clinical symptoms. Therapies aiming at alleviating these proteinopathies are currently being tested in other indications (BIIB100 for TDP-43 pathology in ALS; BIIB080 for Tau pathology in AD, see respective paragraphs above). They might also be tested for the treatment of FTD patients, if the according ALS or AD studies are positive. Another prerequisite would be the availability of PET imaging and respective tracers that allow to reliably identify the underlying proteinopathy. The three most frequently mutated genes in **genetic FTD** are *C9Orf72*, *GRN*, and *MAPT* (with an autosomal dominant inheritance in all three cases). As described before, ASOs downregulating *C9Orf72* or *MAPT* (both predominantly causing a GoF effect when mutated) are currently being tested in C9-ALS and sporadic AD patients and may be tested in C9-FTD cohorts next (see above). Mutations in *GRN* cause FTD by a LOF and aggravate TDP-43 inclusion pathology. An AAV9-based gene reconstitution therapy for GRN-FTD is currently developed towards a phase 1/2 trial (Fig. 1E).

### Polyneuropathies

Hereditary amyloidosis is caused by GoF mutations in the *ATTR* gene leading to an abnormal, aggregation-prone TTR protein that is deposited in amyloid aggregates. The amyloidosis causes cardiomyopathy and/or PNP, also called ***familial amyloid PNP*** (FAP). Two gene specific therapies based on RNA interference, Inotersen and Patisiran, have been developed and approved for the treatment of PNP caused by *hATTR* amyloidosis. Inotersen is an ASO that is administered s.c. once per week, while Patisiran is a first-in-class siRNA therapeutic that is given i.v. every 3 weeks. They lead to the degradation of the mutant and the wildtype *hATTR* mRNA through RNAse H (Inoteresen) (Fig. 1A) and RISC (Patisiran) (Fig. 1D), respectively. Longer lasting compounds based on the same therapeutic principles (AKCEA-TTR-LRx and Vutrisiran) are currently being tested by the same sponsors in phase 3 trials (NCT04136184 and NCT04153149).

***Giant axonal neuropathy*** (GAN) is a very rare, autosomal recessive childhood onset disease owing to LoF mutations in the *GAN* gene. It encodes the protein Gigaxonin. The mutations cause a progressive accumulation of neuronal intermediate filaments in axons. After a successful preclinical study [37] a phase 1 trial is recruiting patients to test the intrathecal administration of an AAV9 vector to deliver a functional copy of the *GAN* gene (NCT02362438) (Fig. 1E).

Duplications of the *PMP22* gene cause the most prevalent subtype of CMT, ***CMT1A***. Zhao et al. have developed an ASO to downregulate *PMP22* mRNA. After s.c. administration the ASO results in restoration of myelination and improvement of electroneurographic parameters in a mouse model based on overexpression of he human *PMP22* gene [38] (Fig. 1A). A clinical trial has not yet been announced. Further, the upregulation of neurotrophin-3 (NT-3) has been shown to lead to a remyelination in CMT1A mouse models and patients [39]. A gene transfer using an AAV1 vector has shown good efficacy in *PMP22* mutant mice [40]. A clinical trial in CMT1A patients is underway (NCT03520751) (Fig. 1E). Preclinical studies testing gene reconstitution therapies have also been successful in mouse models of other CMT types [41].

### Muscle diseases

***Muscle dystrophy*** is a X-linked genetic myopathy caused by mutations in the dystrophin gene *DMD*. Depending on the residual function of the protein product, mutations either lead to the more severe phenotype of Duchenne muscular dystrophy (DMD) (complete LoF) or the milder form of Becker muscular dystrophy (partial LoF). Two ASO therapeutics (Eteplirsen and Golodirsen) have been approved for the treatment of DMD in 2016 and 2010, respectively, by the FDA, while the European authority EMA rejected their approval for reasons discussed below [42]. Eteplirsen and Golodirsen, given s.c. or i.v. weekly, cause a skipping of exon 51 or 53, respectively, of the Dystrophin pre-mRNA. This strategy results in a shortened instead of an otherwise unfunctional protein (Fig. 1B). Both ASOs are restricted to DMD patients with mutations in exon 51 or 53, respectively. However, severe shortcomings of the clinical studies that led to their approval and serious SAEs have raised significant doubts about their efficacy and safety [43, 44]. The FDA has instructed the responsible company to provide more robust evidence for the clinical effectiveness of both ASO therapeutics in post marketing studies by 2021 (Eteplirsen) and 2023 (Golodirsen), respectively. Multiple other ASOs that lead to skipping of various *DMD* exons are being tested in clinical trials [45]. Beyond the aforementioned ASO therapeutics, the small molecule Ataluren has been approved for treatment of patients with nonsense *DMD* mutations, which produce a premature stop codon, after showing some clinical benefit [46]. Ataluren causes the ribosomal readthrough of mRNAs with a premature stop codon and consequent translation of the complete protein. Further, multiple AAV-based i.v. gene transfer therapies have been developed and are being tested in phase 1/2 trials in DMD patients (see Table 1) (Fig. 1E). Since the *Dystrophin* gene exceeds the packaging capacity of AAVs it has been shortened to the essential domains in these cases, called micro- or mini-dystrophin-genes.

***Limb-girdle muscular dystrophy*** (LGMD) is a slowly progressive, symmetric, proximal myopathy with onset in childhood or adolescence. It is caused by mono- or biallelic LoF mutations in various genes encoding sarcoglycans, which tie the intracellular cytoskeleton to the extracellular matrix in muscle tissue. According to the mode of inheritance it is classified into LGMD1 (autosomal dominant) and LGMD2 (autosomal recessive). After successful preclinical studies in mice [47, 48] AAV gene reconstitution therapies that supply healthy copies of the mutated genes have been developed and are being tested in phase 1/2 trials for the autosomal recessive LGMD2 forms D and E (NCT01976091 and NCT03652259 (Fig. 1E).

***Pompe disease*** is a progressive myopathy and autosomal recessively inherited disorder caused by biallelic LoF mutations in the *GAA* gene. *GAA* encodes the lysosomal acidic alpha-glucosidase. Respective mutations lead to lysosomal accumulation of glycogen. After promising results from preclinical mouse studies [49], AAV-mediated expression of *GAA* in hepatocytes by a single i.v. infusion of the viral vector is now tested in Pompe disease patients in phase 1/2 trials (NCT04093349 and NCT03533673) (Fig. 1E). Perspectively, ASOs reducing glycogen synthesis, which are currently being developed for the treatment of Lafora disease, might be a general therapeutic option for glycogenoses.

***Centronuclear Myopathy*** (CNM) is a group of congenital myopathies characterized by abnormal localization of the nucleus in the center of muscles cells. Mutations in several genes have been made responsible for CNM. The most severe from is X-linked CNM (XL-CNM; syn. Myotubular myopathy) is caused by LoF mutations in the *MTM1* gene, while autosomal dominant CNM (AD-CNM) is mostly caused by GoF mutations in the *DNM2* gene. *Mtm1*-KO causes an overexpression of DNM2 and systemic administration of an ASO downregulating *Dnm2* mRNA prevented and reverted myotubular myopathy in *Mtm1*-KO mice [50]. Consequently, a consortium is testing its ASO candidate IONIS-DNM2–2.5_Rx_ (DYN101) that is administered i.v. in patients with centronuclear myopathies caused by mutations in either *DNM2* or *MTM1* (NCT04033159). The study is currently in phase 2. Further, Audentes Therapeutics is testing an AAV8-delivered replacement of the *MTM1* gene (AT132) by single dose i.v. administration in patients with XL-CNM in a phase 1/2 study (NCT03199469). Therapeutic efficacy has already been shown in *Mtm1*-KO mice and XLMTM dogs before. An interim analysis has yielded promising results [51].

### Other neurological indications

**Leber hereditary optic neuropathy** (LHON) is a maternally inherited mitochondrial disease characterized by the degeneration of retinal ganglion cells and their axons leading to vision loss. It is caused by LoF mutations in the genes *ND4*, *ND1* and *ND6* encoding the mitochondrial NADH dehydrogenase proteins. AAV transfer of a healthy *ND4* gene copy after intraocular administration prevented retinal ganglion cell degeneration and preserved visual function in a LHON rat model [52]. GenSight Biologics is testing an AAV2 gene therapy delivering a *ND4* gene copy in a phase 3 trial in LHON patients with *ND4* mutation (NCT02652780, NCT02652767, NCT03293524) (Fig. 1E).

***Neuronal ceroid lipofuscinoses*** (CLN) a group of rare rare, childhood-onset and fatal generally autosomal recessive genetic neurodegenerative lysosomal storage diseases caused by mutations in various genes (CLN1–7). The disease is characterized by symptomatic epilepsy and progressive decline of cognitive and motor functions. After successful preclinical studies in animals [53] trials for the different CLN types testing intrathecal or intracranial administration of single-dose AAV-based gene delivery of the *CLN2*, *CLN3* or *CLN6* gene have been launched by various sponsors and are currently ongoing (CLN2: NCT01414985, NCT01161576; CLN3: NCT03770572; CLN6: NCT02725580).

***Fabry Disease*** is a X-linked genetic lysosomal storage disorder caused by mono- or biallelic LoF mutations in the *GLA* gene and affects men and women. The mutations lead to a deficiency of the alpha-galactosidase, which causes ubiquitous accumulation of glycosphingolipids in lysosomes. This leads to multi-organ dysfunction and polymorphic symptoms, amongst others neuropathy and strokes. After successful preclinical studies in mouse models of Fabry disease [54, 55], Freeline Therapeutics and Sangamo Therapeutics are already testing i.v. single dose AAV gene replacement therapies in phase 1/2 trials in Fabry disease patients (NCT04046224 and NCT04040049) (Fig. 1E).

## Risks and challenges of gene-specific therapies

Although optimism regarding gene-specific therapies is justified, we may not forget that they are still in their infancy and must be considerably improved in many aspects. In the following, we outline the major challenges and risks of current gene selective approaches.

### Drug delivery, expression control and cell selectiveness

Most ASOs applied or tested in CNS disorders presently need to be administered intrathecally. This is not only stressful for the patient but also an infrastructural and financial burden, in view of the many ASO therapies coming that are administered via this route. Therefore, the success of ASO therapies critically depends on the development of suitable vector platforms and conjugate substances capable of sufficiently penetrating brain or muscle tissue after systemic administration. Depending if the target RNA shall be repressed ubiquitously or only in certain cell types the vector or conjugate ideally would also confer a selective tropism. Niche companies are increasingly addressing this matter. The need for improved bioavailability and cell selectiveness also applies to AAV gene transfer therapies. Another significant concern of current gene transfer therapies is that the number of gene copies transferred to a cell is uncontrolled leading to unphysiological up- or downregulation of the target gene, which can be neurotoxic itself in the long run. In addition, it is not precisely known which cell types the AAV infects, and which cell types benefit from the gene transfer, especially when the endogenous version of the delivered gene is normally not expressed in these respective cells. Moreover, current gene transfer therapies use aggressive constitutive promoters instead of tightly regulated endogenous ones. To allow for long term safety, future gene transfer therapies should allow for inducible regulation of the transferred gene in case of relevant adverse effects.

### Long-term complications

Without disrespecting the revolutionary therapeutic effect of gene specific therapies, it must be noted that their current shortcomings are likely to only incompletely compensate the genetic defect and cause long-term complications for various reasons:

1. The disease mechanism is incompletely targeted, e.g. if the pathomechanism is (1) a combination of GoF and LoF or (2) a GOF resulting from repeat expansion. The first constellation would require a selective downregulation of the mutant allele and an upregulation of the healthy one at the same time. However, the majority of current gene silencing approaches are unselective and might exacerbate a concomitant LoF. Repeat expansion mutations lead to toxic RNA transcripts from both the sense and the antisense DNA strand. The repeat RNAs again are translated into toxic repeat proteins. Therefore, an ideal therapy would target both sense and antisense strand of the repeat expanded allele. The observation that repeat expansion can also lead to a relevant LoF at the same time makes this matter even more challenging. On the other hand, allele selective strategies for every single mutation would hardly be affordable and in most instances there would not be enough carriers of the same mutation for a clinical trial.
2. The gene defect is “overcorrected” by the therapy, meaning that gene replacement or gene silencing leads to an unphysiological (maybe neurotoxic) over- or underexpression of the healthy (endogenous or exogeneous) allele in a proportion of cells. Current gene delivery therapies lack expression control. So transfected cells receive varying numbers of exogenous gene copies, resulting in hardly predictable expression levels of the protein product or the regulatory RNAi, respectively. The problem of uncontrolled and unphysiological expression levels is further increased by use of exogenous, strong and constitutively active gene promoters instead of gene specific and conditional endogenous ones.
3. Gene specific therapies can trigger immune responses or be toxic in cell types that should not be targeted. A current example is the dorsal root ganglia inflammation after intrathecal administration of Onasemnogene Abeparvovec-xioi in non-human primates.
4. The gene specific therapy reaches CNS target region(s) only incompletely. Lowered target protein concentrations in the CSF (as observed in the interim analyses of ASO studies targeting SOD1 and HTT expression) are promising, but do not necessarily indicate successful target engagement in the desired neuronal population.
5. Untreated tissues become clinically relevant. Many disease genes (e.g. including *SOD1*, *C9Orf72* and *SMN1*) are ubiquitously expressed, however, most current gene specific therapies only incompletely target a limited number of organs.

### The dilemma of genetic testing

Effective gene specific therapies will become available for more and more disorders soon. Therefore, genetic testing is going to have an increasing therapeutic implication. While it is unquestionable that newborns should be screened for treatable infantile onset genetic diseases (such as SMA), many questions related to genetic testing of adult onset genetic diseases for which gene-specific therapies exist remain unresolved: Who shall be tested – only members of families with a known genetic disease or also sporadic patients without a family history? Who shall be treated? Many patients without family history carry a rare or unique variant in a disease gene that is of unknown significance. The disease mechanisms of most ALS genes are still incompletely understood making it largely impossible to evaluate the pathogenicity of a variant by a specific in vitro assay. Should patients bearing variants of unknown significance be treated with a gene specific therapy, when available – also in view of the currently tremendous costs of these therapies? Also, it is not clearly defined yet when treatment should be started in an asymptomatic carrier of a pathogenic mutation – even if it is known how penetrant and how variable the onset of the specific mutation is. Viewing the incalculable long-term adverse effects of gene selective therapies – do we harm more than we help when we start therapy too early in asymptomatic risk gene carriers? Development of biomarkers reliably indicating the onset of a prodromal disease phase may ease the decision when to start a therapy in disease gene mutation carriers. In any case, these delicate treatment decisions should be met by an expert of both the respective disease and neurogenetics.

### Need for disease specific biomarkers

A slow or inconstant progression, phenotypical variability and small patient numbers challenge speed, costs and not least statistics of clinical intervention trials of genetic diseases, as long as only clinical measures are used as primary endpoints. Therefore, biomarkers that properly correlate with disease progression and can be used surrogates for clinical endpoints are urgently needed. Now is the time to validate biomarkers (e.g. in natural history studies) – once an effective therapy is approved access to native probes of body fluids will be limited.

## Conclusion

Gene-specific therapies targeting a disease gene or disease mechanism are going to revolutionize the treatment of the various genetic and sporadic neurological diseases. However, most of these therapies must be considerably optimized to increase the safety of long-term use and the ease of application. The neurological departments have to anticipate the infrastructural and personal requirements in order to be prepared for the increased demand of genetic counselling and the wave of newly approved therapeutics in in the foreseeable future.

## Data Availability

Not applicable.

## Abbreviations

AAV: Adeno-associated virus
ALS: Amyotrophic lateral sclerosis
AD: Alzheimer disease
ASO: Antisense oligonucleotide
A-syn: Alpha synuclein
CRISPR: Clustered Regularly Interspaced Short Palindromic Repeats
DMD: Duchenne muscular dystrophy
FAP: Familial amyloid PNP
FTD: Frontotemporal dementia
GAN: Giant axonal neuropathy
GoF: Gain of (toxic) function
HD: Huntington’s disease
iPD: Idiopathic Parkinson’s disease
LGMD: Limb-girdle muscular dystrophy
LHON: Leber hereditary optic neuropathy
LoF: Loss of function
miRNA: Micro RNA
MR-guided: Magnetic Resonance Imaging-guided
PD: Parkinson’s disease
PNP: Polyneuropathy
shRNA: Small hairpin RNA
siRNA: Small interfering RNA
RISC: RNA-induced silencing complex
RNAi: RNA interference
SAE: Serious adverse effect
SMA: Spinal muscular atrophy
TALEN: Transcription activator-like effector nuclease
TTR: Transthyretin
ZFN: Zinc-finger nuclease
